# Climate change hopefulness, anxiety, and behavioral intentions among adolescents: randomized controlled trial of a brief “selfie” video intervention

**DOI:** 10.1186/s13034-025-00872-x

**Published:** 2025-02-22

**Authors:** Laelia Benoit, Sarah R. Lowe, Isaiah Thomas, Doron Amsalem, Andrés Martin

**Affiliations:** 1 Yale Child Study Center, Yale School of Medicine, 230 South Frontage Road, New Haven, CT 06520-7900 USA; 2 Secteur de Psychiatrie de l’enfant et de l’adolescent, Hôpitaux Paris Est Val-de-Marne, 94102 Vincennes, France; 3 School of Public Health, Yale University, New Haven, CT 06520 USA; 4 Beth Israel Deaconess Psychiatry, Harvard Medical School, Boston, MA 02215 USA; 5 Vagelos College of Physicians and Surgeons, Columbia University, New York, NY 10027 USA

**Keywords:** Climate change, Mental health, Behavior, Communication, Video

## Abstract

**Aim:**

We tested the utility of showing “selfie” videos to increase adolescents’ climate change hope, agency, and behavioral intentions, and to decrease their climate anxiety.

**Methods:**

We conducted a randomized controlled trial among healthy volunteers, ages 14 to 18, enrolled through a crowdsourcing platform. We randomly assigned participants (N = 1039) to view one of three 110-s-long video interventions featuring the same adolescent protagonist: positive (depicting an action-oriented stance); negative (defeatist stance); and control (neutral stance and unrelated content). The primary outcome was climate change hope; secondary outcomes were climate change anxiety, behavioral intention scales, and 100-point sliders about hopefulness and agency.

**Results:**

Viewing positive “selfie” videos proved effective among adolescents in increasing hopefulness and a sense of agency regarding climate change (< 0.001), but not in decreasing climate anxiety or increasing intentions to engage in pro-environmental behaviors.

**Conclusion:**

Brief video-based interventions featuring adolescent protagonists showed potential to increase hope and agency regarding climate change. While this single exposure did not directly affect anxiety levels or behavioral intentions, future research should examine whether repeated exposure and different "doses" of such interventions might influence these outcomes. The ubiquity and reach of social media hold promise to scale these inexpensive and specifically tailored interventions.

**Supplementary Information:**

The online version contains supplementary material available at 10.1186/s13034-025-00872-x.

## Introduction

### The mental health impact of climate change on youth

Children and adolescents of present and future generations are among the groups whose mental health will be disproportionately affected by climate change [67, 71]. The impact of climate change-related disasters on young people’s mental health is clear [3, 4] and so is the cumulative burden of climate change on their health [1, 16, 27]. Adolescents have been shown to experience a range of negative emotional responses to climate change, including anger, guilt, terror, shame, anxiety, and despair. Climate change anxiety, comprised of negative emotional, cognitive, and behavioral responses to concerns about climate change, has received increasing attention in recent years. Intense anxiety can sometimes lead to eco-paralysis [49], but research increasingly shows that climate anxiety often serves as a catalyst for pro-environmental engagement and action [8, 13, 30, 33, 44]. This anxiety can be adaptive when accompanied by hope and coping strategies, potentially motivating individuals to address environmental challenges.

It is important to acknowledge that some climate change anxiety is a natural and legitimate response to ecological loss [22, 23], increasingly experienced by children and adolescents [21], and which does not imply a mental illness [18]. Even if climate anxiety can be adaptive and help address the climate threat [64], in the long term climate concerns can lead to excess psychological stress [42, 55], which can in turn increase health problems, according to the stress-vulnerability model of health [53]. In 2021, Hickman et al. found that 84% of young people aged 16–25 years are at least moderately worried about climate change (CC) or global warming (GW),with 59% being very or extremely worried and over 50% feel sad, anxious, angry, powerless, helpless, and guilty [29]. Over 45% said their feelings about climate change negatively affected their daily life and functioning. Respondents’ climate anxiety and distress were significantly correlated to perceived inadequate government response and associated feelings of betrayal [29]. Adolescents are more likely to express enduring empathy for the natural world [39] and make decisions readily when facing moral dilemmas: whereas adults often display cognitive dissonance about climate change, young people are more likely to become angry and demand moral clarity [28].

### Adolescents as agents of change

Adolescence is a pivotal developmental stage that makes this age group particularly relevant for climate change interventions. Brain maturation and hormonal changes during this period heighten emotional sensitivity and reactivity [11], amplifying both distress about environmental threats and motivation for action. Adolescents exhibit distinct cognitive patterns, including a lower tolerance for cognitive dissonance compared to adults, driving them to align their understanding of problems with corresponding actions [68]. While adolescents have limited access to traditional power structures and systemic change mechanisms, they exhibit a strong desire for autonomy and agency. This stage is also characterized by heightened sensitivity to peer influence, with peers playing a central role in shaping identity, values, and behavior [34]. Adolescents are more likely to trust and relate to content created by peers, as it resonates with their need for social connection and belonging (Slagter, 2023). Moreover, adolescents are actively forming their identities, exploring values, goals, and social roles. Peer-generated content often reflects shared experiences and aspirations, making it particularly impactful. During this stage, they also develop a deeper understanding of abstract concepts like justice and global responsibility, aligning with messages that foster collective agency [36]. This combination of heightened emotional engagement, reduced cognitive dissonance tolerance, a desire for empowerment, peer influence, identity formation, and emerging moral reasoning, presents unique challenges and opportunities for effective climate change communication and intervention.

### Challenges of climate anxiety and eco-paralysis

Managing climate anxiety is an important challenge. For example, intense fear, especially when combined with guilt, can leave one feeling paralyzed and unable to engage in constructive climate-supportive action. The psychological defense mechanisms of suppression, distraction, and guilt are unhelpful in dealing with the climate crisis. Such mechanisms account for decades of misguided communication about climate change featuring guilt-inducing images, further entrenching the problem [59]. Empirical studies have consistently demonstrated the complex psychological effects of climate change anxiety on behavior, particularly the dual outcomes of eco-paralysis and pro-environmental engagement. Van Zomeren et al provided experimental evidence showing that intense fear related to climate change can lead to both constructive action and emotional avoidance, contingent on coping mechanisms [63]. Similarly, Chen found that fear appeals can effectively motivate pro-environmental behaviors but may induce paralysis when self-efficacy is low [14]. Innocenti et al. highlighted the mediating role of general self-efficacy, showing that individuals with low self-efficacy are more prone to eco-paralysis despite heightened climate anxiety [30]. Complementing these findings, von Gal et al. emphasized that fear and guilt, when unmoderated by coping strategies, exacerbate eco-paralysis, impeding constructive climate action [65]. Together, these studies underline the need to address self-efficacy and adaptive coping to mitigate the paralyzing effects of climate-related emotions.

### Maladaptive responses to youth climate concerns

Young people’s environmental concerns have not always been met with understanding. In a previous study based on American newspapers’ discourses regarding youth and CC/GW, we described the immature ways in which adults respond to young people’s climate anxiety and actions [9]. Through the framework of *childism* [73]*,* we described these critiques of young people as a defensive stance against climate accountability. We posit an alternate approach, informed by the principles of existential psychology [72]. Climate change confronts people with all four fundamental existential concerns: *death* (through ecological loss and mortality), *freedom* (through choices about environmental impact), *isolation* (in navigating individual versus collective responsibility), and *meaninglessness* (in questioning purpose amid environmental crisis). Most people avoid these challenging existential questions due to inadequate coping mechanisms [9]. However, positive messaging can help frame these concerns constructively, fostering agency rather than paralysis. This approach can inform healthier and more productive responses from adults as they seek truthful yet supportive responses to address legitimate ecological threats that will disproportionately affect future generations. Similarly, Schwartz et al. (2002) have shown that environmental activism may buffer young people's climate anxiety. The contrasting negative messaging condition reflects a common but maladaptive response to existential threats: *resignation* and *defeat*.

### The gap between awareness and action

Although most adolescents do not believe that young people’s concerns about climate change are being suitably addressed, fewer are taking action to reduce their own carbon footprint. Most of them rarely or never discuss the issue with family and friends, and only 14% believe that they have learned enough at school about climate change [26]. This gap between belief and behavior may be due to individual actions seeming unimpactful, leading to feelings of disempowerment [31], helplessness, and demoralization [51]. Experts in the field of climate change and child mental health emphasize the need for researchers to prioritize the design of interventions to mitigate the psychological impact of the climate crisis on young people [69].

### Study rationale and objectives

Youth may also experience positive emotions about climate change– namely, hope. Our project aims to harness traditional and social media outreach efforts to create constructive and empowering messaging that helps young people channel their climate anxiety into climate action. The purpose of this study is to test the impact of watching brief video-based interventions designed in the style of a peer’s TikTok “selfies” (self-filmed testimonials) among adolescent viewers to: (1) Reduce climate anxiety; (2) Increase behavioral intentions for climate action; (3) Compare messaging styles; and (4) Examine the impact of viewers’ gender and race as independent factors on the outcomes of interest. We hypothesized that viewing positive messaging would lead to greater climate hope and sense of agency than neutral or negative messaging.

For this study we used brief “selfie” video methodology. This design enables scalability since brief videos can be shared on social networks. Selfies are inexpensive to produce and feature protagonists who master the language and codes of young people. We used a similar methodology in prior works aimed at decreasing stigma around depression and increasing treatment-seeking intentions among adolescents [7], including adaptations to the unique experiences of depression among Black adolescents (such as anti-Black racism) [40] and transgender and gender-diverse youth (transphobia)[5].

## Methods

### Intervention

We used three brief stimuli videos for participants to view, each designed in the format of a TikTok "selfie" (self-recorded testimonial) and performed by the actor-protagonist, a 16-year-old adolescent girl. To be clear, participants were assigned to watch these pre-recorded videos; they did not create videos themselves. We had previously demonstrated the equivalence between selfie and professionally recorded videos in two non-inferiority RCTs, one conducted with adults, another with adolescents [6]. The selfie format was chosen to maximize authenticity and relatability for adolescent viewers, as it mirrors the self-recorded video content they commonly encounter on social media platforms.

Stoknes [59] emphasizes that many climate change communications focus on highlighting its severe consequences to raise public awareness but often lack actionable strategies for individual and collective engagement. He argues that this approach, when not paired with concrete examples of action, can result in feelings of despair and paralysis. To address this issue, we developed three versions of a selfie-video intervention, each beginning with a brief overview of the negative impacts of climate change. For this study, the actor describes her stance and behaviors concerning CC/GW. In a first version (positive), her stance is can-do and action-oriented, emphasizing individual and collective actions she has already undertaken (e.g., “we got the mayor to add more bike lanes, and that’s just the beginning. We can be the change. We can do this”). In a second selfie (negative), she presents with a pessimistic and defeatist stance (e.g., “ It’s like, nobody cares. I don’t know what to do”). In a third video (control), the same actor summarizes one of her favorite books. The third video makes no reference to CC/GW or related topics.

We hired the professional actor through the Youth Simulated Participant Program of the Child Study Center, Yale School of Medicine. We trained, debriefed, and compensated the actor in keeping with best practices for standardized patients [19].The three stimulus video clips each had a runtime of 1′50″ (110 s), and are available for viewing through the URL links in Appendix 1.

### Recruitment procedure, participants, and ethics approval

We recruited participants using CloudResearch [12], a crowdsourcing platform widely used in social science with specific experience enrolling minors. We included only English-speaking youth, 14 to 18 years old, and living in the United States. We assigned participants randomly to one of the three conditions on a balanced ratio (negative, control, positive), stratified by gender (female or male) and race (Black, White, or other). For the purposes of randomization, individuals who selected “non-binary”, “other”, or “prefer not to disclose” as their gender (6% of total sample), and individuals who selected “other” or “prefer not to disclose” as their race (23% of total sample), were randomly assigned them in equal parts to the female / male, or to the Black / White dichotomous groups, respectively.

To ensure the sample’s integrity, we used several accepted methods of quality control. First, we used an open-ended question asking for age and only allowing a two-digit number as valid. Second, we included a CAPTCHA question (Completely Automated Public Turing Test to Tell Computers and Humans Apart) to prevent contamination by bots. Third, we added timers before enabling the “next” button, to ensure participants had enough time to read the instructions (7-s minimum) and watch the video (110-s minimum). Fourth, we excluded individuals who tried to answer the survey more than once, completing the assessment in an unusually short amount of time, were located in GPS locations outside of the U.S., or possessed unusual IP addresses. Finally, we excluded participants who failed a validity question with a forced, single response (“Mark ‘very much’ from the options below.”).

### Ethics declaration

In accordance with the principles of the Declaration of Helsinki, this study was approved by the Yale Human Investigations Committee / Institutional Review Board (Protocol # 2000028980, MOD 2000032064) and was pre-registered in ClinicalTrials.org before starting data collection in May 2022 (ID: NCT05372705). Volunteers accessed the study through WiFi-enabled devices, whether mobile or laptop, and we compensated them $3.50 for their time. In the first screen, respondents reviewed an informed assent that waived parental consent, as approved by the institutional review board (IRB). Assenting adolescents were directed to a secure platform for data collection (Qualtrics; Provo, UT).

### Instruments

We assessed attitudes and behavioral intentions related to CC/GW using two empirically validated scales: the Climate Change Hope (CCH) scale, and the Climate Change Anxiety (CCA) scale, as well as a custom measure of behavioral intentions developed specifically for this study.

The CCH [37] is an adaptation for adolescents of an earlier scale measuring a similar construct [45]. It consists of 11 items and 3 subscales: personal will and way (PW, 4 items),collective will and way (CW,5 items), and lack of will and way (LW, 3 reverse-scored items.) The CCH is rated on a 7-point Likert scale ranging from strongly disagree (1 to strongly agree 7). Scores comprise the sum of its item scores, with higher score indicating more hopefulness, except for the reverse scored LW. The CCH subscales have adequate psychometric properties: Cronbach’s α of 0.92 and 0.82, respectively. We used the CCH total score as primary, and its 3 subscales and 11 individual items as secondary endpoints.

The CCA [17] is a measure of anxiety related to CC/GW. It consists of 13 items and 2 subscales: cognitive emotional impairment (CEI, 8 items), and functional impairment (FI, 5 items). The CCA is rated on a 5-point Likert scale ranging from never (1) to almost always (5). Scores comprise the sum of its item scores, with higher scores indicating more anxiety. The two CCA subscales and overall scale have adequate psychometric properties: Cronbach’s α of 0.68, 0.72, and 0.78, respectively. We used the overall scale, subscale, and individual item scores as secondary endpoints.

As a third outcome, we created an 8-item measure of behavioral intentions (BI) regarding CC/GW, each one with the same stem question: “How likely is it that over the next six months you will…?”. Items include “Educate myself about individual actions I can take to address CC”, “Participate in nature conservation efforts”, “Lead or organize a CC protest or rally” among others. Possible responses range from “Not at all likely” (1) to “Extremely likely” (5). The overall score is the sum of the items, with higher scores indicating greater behavioral intentions. The scale had a Cronbach α of 0.76 in this study.

In our previous studies we used sliders and single word approaches as exploratory measures [5, 7, 40]. In this study, we used two sliders to gauge attitudes regarding CC/GW on a scale from 0 to 100: 1) hopefulness about the future: “I feel hopeful about climate change”, and 2) sense of agency to make a positive impact: “I can help address climate change”. Finally, we asked for the single word that first came to mind about CC/GW. We repeated all outcome measures before and after the randomized selfie video intervention.

### Data analysis

We used Pearson’s chi-square and one-way ANOVA to compare demographic variables between groups. For the primary outcome measure (CCH total score), we used univariate ANOVA to compare mean change in scores after the intervention (i.e., endpoint minus baseline values) across the three intervention groups (negative, control, and positive). We used post hoc Tukey HSD tests to determine pairwise differences between interventions. We used the same analytic approach for each of the secondary outcome measures. Next, we analyzed all outcomes stratified across two baseline characteristics: 1) Belief that CC/GW is related to human action (yes/no), and 2) Having experienced the impact of CC/GW, either personally or among family members (yes/no). Finally, for item-level analyses of the CCH, CCA, and BI, we use paired t tests to compare change from baseline to endpoint in each of the three intervention groups. We used the Bonferroni correction for the 34 t-tests, considering as significant only those results with p values below the p < 0.001 threshold (i.e., 0.05 / 50, adjusted for up to 50 comparisons). We conducted all statistical analyses using IBM SPSS software, version 26.0 (Armonk, NY).

## Results

**Fig. 1:**
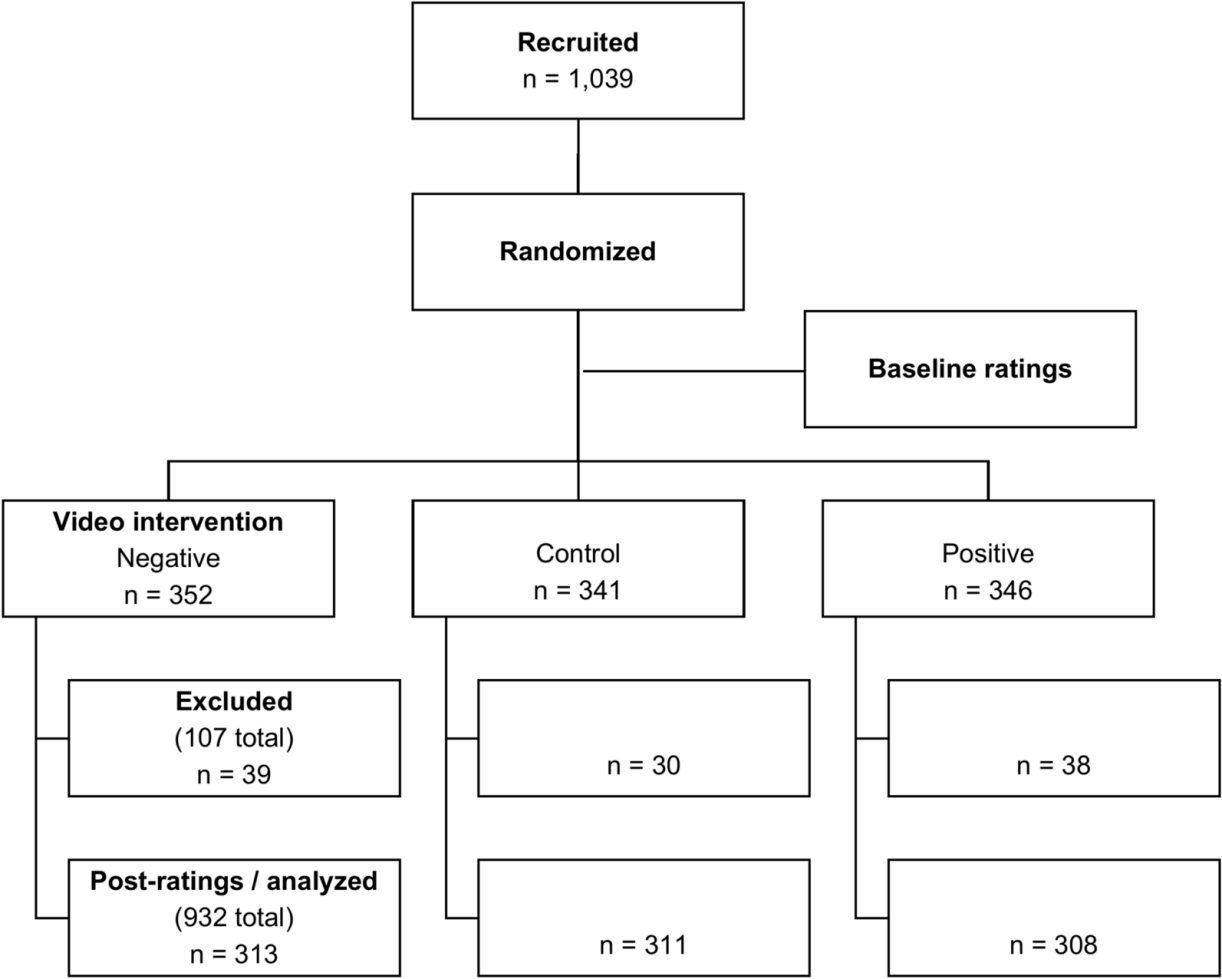
Study flow chart

We screened 1,299 individuals and excluded 260 (20%) who did not meet inclusion criteria. We recruited 1,039 participants, who completed baseline assessments before being randomly assigned to one of the three intervention groups. Participants took a median of 8.7 min to complete the tasks (interquartile range, 6.9 to 11.3 min; p > 0.05 across groups). We excluded from analysis 107 individuals (10%) who completed endpoint ratings, but failed the embedded validity question, yielding a total of 932 participants, evenly divided across the three video conditions. Figure 1 shows the study’s flow, and Table 1 summarizes the baseline demographic characteristics of the analyzed sample.

**Table 1:** Demographics

Group n	Negative 313		Control 311		Positive 308		All 932		Statistic	
	n	%	n	%	n	%	n	%	X^2^/F	p
Age (Mean years/SD)	16.8	1.2	16.9	1.1	16.8	1.2	16.9	1.2	0.40	0.668
Gender									1.29	0.972
Female	149	48	151	49	149	48	449	48		
Male	149	48	141	45	139	45	429	46		
Other/not disclosed	15	5	19	6	20	6	54	6		
Race									2.25	0.895
Black	56	18	58	19	58	19	172	19		
White	184	59	183	59	183	59	550	59		
Other/not disclosed	73	23	70	23	67	22	210	23		
Hispanic/Latino/a/x	80	26	74	24	73	24	227	24	0.57	0.966
Spiritual and/or religious	168	54	178	57	165	54	511	55	1.09	0.579
Climate change/global warming										
Is related to human actions (n = 888)	289	88	298	92	287	88	874	89	2.60	0.272
Have experienced its consequences	111	36	124	40	126	41	361	39	2.20	0.334

There were no differences in demographic characteristics across the three intervention groups (p > 0.05). Male and female genders were evenly distributed, with 54 (6%) of participants identifying as non-binary or preferring not to state their gender. Black participants (n = 72) comprised 4.5% of the overall sample, a lower proportion than in the 2019 US census (14%)[60]. Individuals of a race other than Black or white (n = 210, 23%) primarily self-identified as “mixed or other” (n = 122, 13%), Asian (n = 70, 8%), or Indigenous / Native American (n = 18, 2%). Nearly one quarter of the sample (n = 227, 24%) identified their ethnicity as Latino/a/x or Hispanic.

We found a robust difference in our primary outcome (CCH change from baseline) across the three conditions (F = 13.94, df = 2,931, p < 0.001; Table 2). Positive (P) and negative (N) interventions outperformed control (C; Tukey HSD p < 0.001) but did not differ from one another (p = 0.637). In the hopefulness and agency sliders, Positive outperformed Negative and Control (p < 0.001), which did not differ from each other. Interestingly, while the negative messaging did not increase hopefulness, it outperformed the control condition. We found a smaller change in the CCA and its CEI subscale, where Positive was not significantly different than Control and outperformed Negative (p = 0.24 and 0.036, respectively). We found no change in BI, and a small correlation between the hopefulness slider and BI (r = 0.124, p < 0.001).

**Table 2:** Climate Change Hopefulness scale: item-level change after intervention (n = 932)

Group n	Negative 313	Control 311	Positive 308
Climate Change Hopefulness (CCH)	− 0.17	**− 5.99**	1.14
Personal will and way (PW)	0.72	**− 3.83**	**3.48**
1. I am willing to take actions to help solve problems caused by climate change	0.17	**− 4.78**	− 1.56
2. I know that there are things that I can do to help solve problems caused by climate change	− 0.95	**− 3.16**	0.77
3. I know what to do to help solve problems caused by climate change	2.25	0.00	**7.48**
Collective will and way (CW)	− 0.77	**− 5.96**	2.29
4. If everyone works together, we can solve problems caused by climate change	1.05	**− 5.32**	− 1.28
5. I believe that scientists will be able to find ways to solve problems caused by climate change	0.24	− 2.54	1.68
6. I believe people will be able to solve problems caused by climate change	− 2.85	**− 5.43**	− 1.19
7. I believe more people are willing to take actions to help solve problems caused by climate change	0.83	0.00	**6.58**
8. Even when some people give up, I know there will be others who will continue to try to solve problems caused by climate change	− 1.24	**− 4.39**	1.21
(R) Lack of will and way (LW)	− 0.20	− 2.59	**− 3.30**
9. (R) Climate change is beyond my control, so I won’t bother trying to solve problems caused by climate change	− 0.38	− 0.86	− 1.54
10. (R) The actions I can take are too small to help solve problems caused by climate change	0.37	**− 3.41**	**− 3.32**
11. (R) Climate change is so complex we will not be able to solve problems that it causes	− 0.44	− 1.82	**− 2.62**
Hopefulness (slider)	1.17	2.00	**11.42**

Paired t values compare differences between baseline and endpoint scores;

Values in bold are significant at p < 0.001

Higher scores indicate more hopefulness, except for items indicated as reversed (R)

In item-level analyses, we found significant improvements in 6 of 11 CCH items in the Positive group, compared to 2 in the Control, and none in the Negative groups (Table 3). Although we found differences in CCA and BI individual items, they did not reach statistical significance (Table 4).

**Table 3:** Climate change hopefulness scale: item-level change after intervention (n = 932)

Group n	Negative 313	Control 311	Positive 308
Climate Change Hopefulness (CCH)	− 0.17	**− 5.99**	1.14
Personal will and way (PW)	0.72	**− 3.83**	**3.48**
12. I am willing to take actions to help solve problems caused by climate change	0.17	**− 4.78**	− 1.56
13. I know that there are things that I can do to help solve problems caused by climate change	− 0.95	**− 3.16**	0.77
14. I know what to do to help solve problems caused by climate change	2.25	0.00	**7.48**
Collective will and way (CW)	− 0.77	**− 5.96**	2.29
15. If everyone works together, we can solve problems caused by climate change	1.05	**− 5.32**	− 1.28
16. I believe that scientists will be able to find ways to solve problems caused by climate change	0.24	− 2.54	1.68
17. I believe people will be able to solve problems caused by climate change	− 2.85	**− 5.43**	− 1.19
18. I believe more people are willing to take actions to help solve problems caused by climate change	0.83	0.00	**6.58**
19. Even when some people give up, I know there will be others who will continue to try to solve problems caused by climate change	− 1.24	**− 4.39**	1.21
(R) Lack of will and way (LW)	− 0.20	− 2.59	**-3.30**
20. (R) Climate change is beyond my control, so I won’t bother trying to solve problems caused by climate change	− 0.38	− 0.86	− 1.54
21. (R) The actions I can take are too small to help solve problems caused by climate change	0.37	**− 3.41**	**− 3.32**
22. (R) Climate change is so complex we will not be able to solve problems that it causes	− 0.44	− 1.82	**− 2.62**
Hopefulness (slider)	1.17	2.00	**11.42**

Paired t values compare differences between baseline and endpoint scores

Values in bold are significant at p < 0.001

Higher scores indicate more hopefulness, except for items indicated as reversed (R)

**Table 4:** Climate Change Anxiety scale, behavioral intentions, and agency: item-level change after intervention (n = 932)

Group n	Negative 313	Control 311	Positive 308
Climate change anxiety (CCA)	**− 3.56**	**− 6.98**	− 2.46
Cognitive emotional impairment	**− 4.64**	**− 8.13**	**− 3.80**
1. Thinking about climate change makes it difficult for me to concentrate	**− 10.08**	**− 11.34**	**− 8.64**
2. Thinking about climate change makes it difficult for me to sleep	**− 4.72**	**− 7.90**	**− 4.52**
3. I have nightmares about climate change	− 0.30	1.52	− 0.66
4. I find myself crying because of climate change	− 0.12	− 0.86	0.47
5. I think, “why can’t I handle climate change better”?	− 2.41	**− 4.39**	− 3.08
6. I go away by myself and think about why I feel this way about climate change	− 1.37	− 2.56	− 0.31
7. I write down my thoughts about climate change and analyze them	− 0.79	0.13	1.25
8. I think, “why do I react to climate change this way?”	− 0.51	**− 3.34**	0.73
Functional impairment	− 0.84	− 2.84	0.54
9. My concerns about climate change make it hard for me to have fun with my family or friends	− 1.50	− 2.76	0.60
10. I have problems balancing my concerns about sustainability with the needs of my family	**− 3.37**	− 3.53	− 2.04
11. My concerns about climate change interfere with my ability to get work or school assignments done	− 0.87	0.30	0.90
12. My concerns about climate change undermine my ability to work to my potential	0.31	− 1.80	1.51
13. My friends say I think about climate change too much	1.25	0.00	1.30
Behavioral Intentions *(Likelihood over the next six months to…)*	− 0.68	− 2.20	− 0.97
1. Educate myself about individual actions I can take to address climate change	− 2.63	− 0.81	− 1.56
2. Educate myself about actions I can take with other members of my community to address climate change	− 1.11	0.00	− 0.75
3. Raise awareness about climate change (talk with others, use online tools, etc.)	− 0.11	− 1.44	− 1.25
4. Participate in nature conservation efforts (e.g. planting trees)	− 0.23	0.21	-0.36
5. Participate in a climate change protest or rally	− 0.88	− 0.74	− 0.35
6. Join an organization focused on addressing climate change	− 0.89	− 2.61	− 1.05
7. Take on a leadership role in an organization focused on addressing climate change	0.75	− 1.70	− 0.68
8. Lead or organize a climate change protest or rally	1.68	− 0.69	1.44
Agency (slider)	**5.31**	**4.71**	**8.37**

Paired t values compare differences between baseline and endpoint scores

Values in bold are significant at p < 0.001

Higher scores indicate greater anxiety, behavioral intentions, or agency

In stratified analyses across beliefs and impact, we found a more important response to intervention across CCH, hopefulness, and agency outcomes among the participants who considered CC/GW associated with human actions (n = 792, 89%), than among those who did not (n = 96, 11%; Table 5). In a more evenly split division, those who had *not* experienced the impact of CC/GW directly (n = 361, 39%) were more responsive to intervention (i.e., showed greater change on outcome measures) than those who had (n = 361, 61%).

**Table 5:** Outcomes stratified across climate change / global warming beliefs and impact

Group n	Climate change/global warming			
	Are related to human action n = 888		Have experienced its impact n = 932	
	Yes 792	No 96	Yes 361	No 571
Climate change hope (CCH)	10.00***	3.39*	1.27	15.21***
Personal will and way (PW)	12.06***	2.4	6.41**	6.27**
Collective will and way (CW)	11.21***	5.9**	3.78*	14.60***
Lack of will and way (LW)	6.27**	0.61	6.37**	4.11*
Hopefulness	31.89***	4.06*	11.90***	20.13***
Climate change anxiety (CCA)	3.42*	1.36	4.71*	0.88
Cognitive-emotional impairment	3.50*	0.87	3.23*	1.27
Functional impairment	1.42	1.87	4.06*	0.23
Agency	9.69***	0.20	1.70	9.91***
Behavioral intentions	0.42	0.86	0.99	0.20

F values derived through univariate ANOVA comparing negative/control/positive conditions

^*^p < 0.05, **p < 0.01 ***p < 0.001

**Fig. 2:**
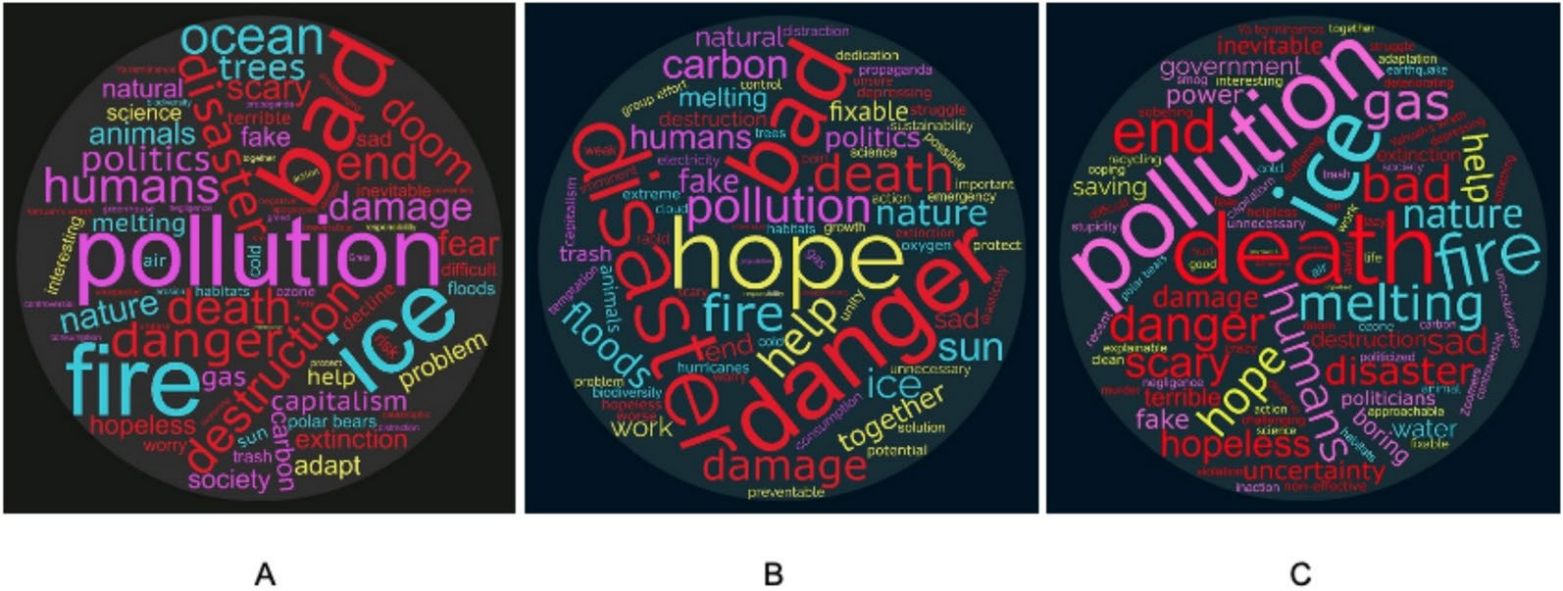
Word clouds of terms that come to mind when thinking of “climate change” or “global warming”. Panel **A**: pre-intervention (positive and negative groups combined, n = 381); **B**: Post-intervention, positive (n = 218); **C**: Post-intervention, negative group (n = 241)

When asked for the single word that first came to mind about CC/GW before the intervention, the most frequent word among all participants was “pollution” (n = 34) in both P and N groups combined (n = 381, Fig. 2A). After the intervention, “hope” (n = 22) was the most frequent word in the P group (n = 218, Fig. 2B), whereas “death” (n = 22) was the most frequent in the N group (n = 241, Fig. 2C). The adjacent word clouds reveal how action-oriented terms (denoted in yellow) increased after positive messaging, even if danger-oriented ones (denoted in red) didn’t necessarily decrease. Words referring to causes of CC (denoted in pink) and to nature (denoted in light blue) remained stable.

## Discussion

This is, to our knowledge, the first study to explore adolescents’ responses to viewing brief “selfie” videos with messaging explicitly related to climate change and global warming. With their short duration and specific formatting, the videos we tested were designed for deployment through popular social media platforms (e.g., TikTok, Instagram Reels, YouTube, Snapchat Stories, or Facebook Stories). We go on to highlight our findings, place them in the context of the literature, and outline the next steps for this line of developmentally attuned environmental effort.

### Climate hope, agency and action

As hypothesized, “selfie” videos with positive messaging proved effective among adolescents at increasing hopefulness and a sense of agency regarding CC/GW. The control condition's notably worse performance on hopefulness measures may reflect the cognitive dissonance created by watching content entirely disconnected from climate change, potentially triggering a sense of absurdity about addressing trivial matters while avoiding crucial environmental concerns. This unexpected finding suggests that even pessimistic engagement with climate change may be preferable to complete disconnection from the issue. Furthermore, since the negative video begins by acknowledging climate change's importance, it may be less demoralizing than experiencing complete disregard for the issue, as represented in the control condition. This result is promising, as hope and coping strategies have been shown to foster environmental engagement among young people [45, 47]. In 12-year-old adolescents, both problem-focused and meaning-focused coping styles are positively associated with measures of environmental engagement [45]. Hope also increases pro-environmental behavior and decreases the risk of reduced wellbeing as a result of climate anxiety [46]. Regarding feelings of agency, Tayne et al. analyzed students’ discourses about climate action: if participants predominantly frame climate action as individual behaviors, change is framed as effective only when many individuals participate. The authors argue that shifting from a discourse focused on individual behavior change to collective and system-oriented change supports hope and agency, as well as social justice [61]. In a previous international qualitative study with young people, we showed that feelings of climate hope and agency are shaped by their social class [15] and by the degree of autonomy granted to young people in their country [62].

### Climate change anxiety

Contrary to our prediction, the positive “selfie” video did not decrease climate anxiety among participants. This result is consistent with a survey by Schwartz et al., which shows that, even if climate change anxiety is correlated with engagement in individual action, individual action alone does not reduce emotional distress. Engaging in collective action, rather than only individual actions, significantly attenuates the association between climate change anxiety and depressive symptoms [54]. In a qualitative analysis of the experience of adults seeking psychotherapy for climate change anxiety, Budziszewska & Jonsson showed that learning to manage their emotions rather than making them disappear were salient aspects of psychotherapy from the patients’ perspective [10]. Existential psychology describes how humans’ awareness of their freedom and responsibility triggers feelings of anxiety [72]. Some lingering ‘decision-making’ anxiety becomes manageable once a person is aware that it is an inherent component of human agency, and therefore responsibility [9]. As such, climate mental health interventions should provide skills to manage anxiety, rather than try to suppress it.

### Behavioral intentions

Behavioral change, including intention toward it, is a more distal outcome than change in perceptions. As such, we were not entirely surprised that a single exposure to positive “selfie” messaging did not increase participants’ intentions to engage in pro-environmental behaviors. This result is moreover consistent with the existence of factors known to moderate adolescents’ environmental behavior. Both adolescents’ behavioral intention and degree of social exposure are associated with their environmental behavior [35, 43]. But adolescents’ pro-environmental behaviors are also influenced by norms within their families [2, 20, 24, 32] and peer groups [20, 66]. Indeed, identifying with others, group participation, reciprocity, and personal norms are more likely to enhance young people’s pro-environmental behavior and activism [32, 66]. Online peer persuasion behavior is also explained by the youth’s own environmental self-efficacy, environmental news consumption, political interest, time spent online, gender, and environmental consumerism [2]. As marketing research shows that repetition increases commercial effectiveness [38, 56], future environmental communication research may assess the impact of a series of videos repeated over time.

If adolescents’ environmental attitudes predict pro-environmental behaviors, this relationship is moderated by their environmental knowledge [43] and the indirect effect of climate education [58]. Stevenson et al. found a causal relationship between climate education and gains in climate change knowledge, but not between climate education and pro-environmental behavior. However, the authors did find support for a path model in which climate change knowledge positively relates to increased climate change concern and hope, and increases in concern and hope predict changes in pro-environmental behavior [58]. Along these lines, the small correlation we found between hopefulness and behavioral intentions suggests that hope could be a mediating factor of environmental behavior.

### Human responsibility

The impact of the “selfie” intervention was attenuated among the relatively small number (n = 96, 11%) of participants who did *not* consider CC/GW to be the result of human activity. From 1976 to 2005, trends among high school seniors showed that across all years “youth tended to assign responsibility for the environment to the government and consumers rather than accepting personal responsibility” [70]. Similarly, de-emphasizing the seriousness of climate change has negative association with engagement [46]. Climate skepticism in adolescents can be explained by different factors: values, knowledge, conservative political orientation, gender, media use, societal powerlessness, social influence from parents and peers, and a perception that the fate of the environment lies in the hands of powerful others [48]. By contrast, perceptions of self-efficacy and intergenerational obligation, that is, believing that all generations should take action, predict the likelihood of engaging in environmental behavior and educational commitment [52].

### Limitations and next steps

This research has limitations. The sample is US-based, English-speaking only, and crowdsourcing platforms are not usually representative of the general population (e.g., our sample included lower rates of Black participants than the general U.S. population). The recruitment material mentioned climate change, which may have enriched the enrollment of participants with a greater interest in climate. There was limited statistical power to examine differences across more refined demographic groups, and a low internal consistency of some measures. This study did not include a follow up assessment. As such, a one-time exposure to brief “selfie” videos is a proof-of-concept rather than an intervention. Repeated exposure to tailored and updated messaging that is part of a broader public health initiative will be required, particularly to sustain the engagement of youth in this process [50]. Additional research is needed to explore the developmental trajectory of long-term environmental stewardship and activism from childhood through adulthood [25, 41]. Further studies should assess interventions specifically tailored to engage the minority of young people who express skepticism regarding human responsibility in climate change. In each of these areas, social media platforms using brief “selfie” videos can have much to offer as a vehicle for public health initiatives around climate change: youth-driven and bespoke messaging, low production costs, wide distribution reach, and ability to track metrics. Our findings suggest that future interventions to impact climate health action will need to balance individual withgroup-based messaging, and consider target audiences’ baseline perceptions,

## Conclusion

Brief video-based interventions featuring adolescent protagonists showed potential to increase hope and agency regarding climate change. While this single exposure did not directly affect anxiety levels or behavioral intentions, future research should examine whether repeated exposure and different "doses" of such interventions might influence these outcomes. The ubiquity and reach of social media hold promise to scale these inexpensive and specifically tailored interventions.

## Supplementary Information

Supplementary material 1.

## Data Availability

Research raw data are available from the corresponding author upon reasonable request.
