# Supplementary material for: Climate change hopefulness, anxiety, and behavioral intentions among adolescents: randomized controlled trial of a brief “selfie” video intervention
Source: Child Adolesc Psychiatry Ment Health. 2025 Feb 22;19:13. doi: 10.1186/s13034-025-00872-x (PMC11847372; doi:10.1186/s13034-025-00872-x)
Supplement: Supplementary file 1 — Supplementary material 1. [file 13034_2025_872_MOESM1_ESM.docx]

**Appendix 1: Link to the three videos – Positive – Negative – Control**

https://drive.google.com/drive/folders/1aZ3v93tUHaQWXTDaLZTP3WTX2Y20LaLb?usp=sharing
